# Therapeutic targeting of MALT1 in oncology: Mechanism, inhibitor development, and clinical prospects

**DOI:** 10.1002/ccs3.70066

**Published:** 2026-03-02

**Authors:** Xintao Cao, Peixia Wang, Yuan Ji, Yongliang Sun, Dejiu Zhang

**Affiliations:** ^1^ Department of Discovery Biology Shouyao Holdings (Beijing) Co. Ltd Beijing China; ^2^ National Anti‐ Drug Laboratory Beijing Regional Center Beijing China; ^3^ College of Basic Medical Qingdao Binhai University Qingdao China

**Keywords:** B‐cell malignancies, MALT1, MALT1 inhibitors, NF‐κB signaling pathway, tumor therapy

## Abstract

MALT1, a multifunctional protease molecule, plays a pivotal role in the adaptive immunity by regulating immune cell survival, proliferation and activation through the nuclear transcription factor‐κB (NF‐κB) signaling pathway by scaffold and protease activities. Aberrant activation of MALT1 is implicated in the pathogenesis of hematologic malignancies, particularly diffuse large B‐cell lymphoma, and select solid tumors. Emerging research studies highlight MALT1 inhibitors as promising therapeutic agents for B‐cell malignancies, with several candidates demonstrating preclinical and clinical efficacy. Notably, agents such as safimaltib (JNJ‐67856633) have shown manageable safety profiles and preliminary antitumor activity in early‐phase trials for relapsed/refractory B‐cell malignancies. However, MALT1‐targeted therapy poses a dual challenge: although inhibiting oncogenic signaling and tumor cell proliferation, it also disrupts immunosuppressive Treg function, risking autoimmune toxicity by compromising the tumor microenvironment. This review systematically analyzes MALT1's oncogenic roles across cancers, clarifies inhibitor mechanisms, and evaluates translational challenges and strategic opportunities for precision oncology and combination immunotherapy.

## Graphical abstract

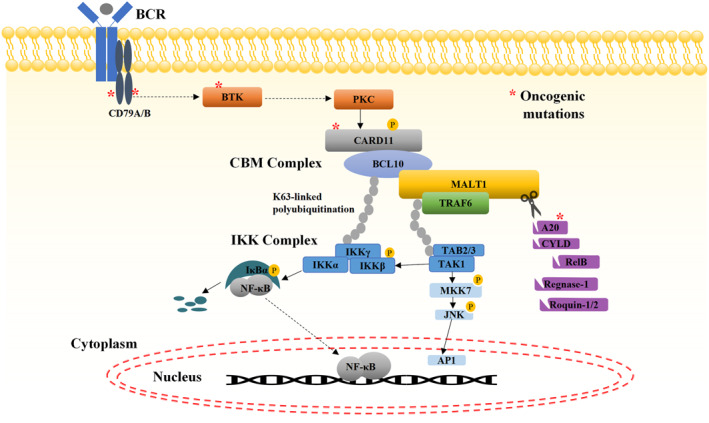

## 1 INTRODUCTION

Mucosa‐associated lymphoid tissue protein 1 (MALT1), also known as paracaspase 1 (PCASP1), is a molecule with protease activity that has attracted considerable attention in tumor therapy. The discovery of MALT1 stems from an in‐depth study of human and murine adaptive immune responses, in which MALT1 plays a key role in B and T cell activation, cytokine production, proliferation, and differentiation.^1^, ^2^, ^3^ These biological processes are essential for maintaining the equilibrium and effectiveness of the immune system. Aberrant activation or expression of MALT1 is closely associated with the onset and progression of a variety of diseases, particularly tumors. In recent years, with the continuous revelation of the mechanism of MALT1 in tumor biology, MALT1 has been shown to finely regulate the nuclear transcription factor‐κB (NF‐κB) signaling pathway through its unique scaffold function and protease activity, thereby impacting the survival and proliferation of tumor cells and the stability of the tumor immune microenvironment.^4^, ^5^, ^6^, ^7^, ^8^ As a result, MALT1 has become a focal point in the study of hematologic malignancies and solid tumors.

The development of MALT1 inhibitors represents an important direction for novel anticancer strategies. With a better understanding of the mechanism of action of MALT1 in tumors, significant progress has been made in the investigation of MALT1 inhibitors. These inhibitors have shown therapeutic efficacy against B‐cell tumors by blocking the protease activity of MALT1 or by interfering with its function in the NF‐κB signaling pathway.^7^, ^9^, ^10^, ^11^ With the ongoing advancement of clinical trials, MALT1 inhibitors are expected to provide major breakthroughs in the field of cancer treatment, offering patients with more innovative and effective treatment options. This article aims to review the mechanism of action of MALT1 in tumors and the latest progress in the development of MALT1 inhibitors, to explore the differences in the role of MALT1 in different types of tumors, and the potential and challenges of MALT1 inhibitors in preclinical and clinical research. We hope to provide readers with a comprehensive perspective on the use of MALT1 and its inhibitors in cancer therapy, and look forward to future research directions.

## 2 OVERVIEW OF MALT1

**FIGURE 1:**
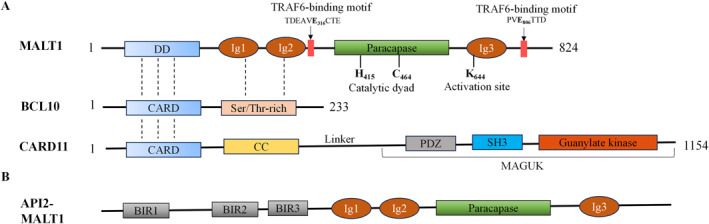
Domain organization of MALT1, BCL10, CARD11 and API2‐MALT1. (A) MALT1 structural organization. The MALT1 protein comprises five functional domains: an N‐terminal death domain (DD), two immunoglobulin‐like (Ig1/2) domains, a paracaspase domain, and a C‐terminal Ig3 domain. Motifs required for binding to TRAF6 are indicated above. Residues controlling protease function are shown below, including the active site residues (C464 and H415) and the monoubiquitination site (K644) required for MALT1 activation. Dashed lines indicate the domains interaction between MALT1 and BCL10, and CARD11. (B) API2‐MALT1 fusion protein consists of three BIR domains of N‐terminal AIP2 and the MALT1 sequence lacking the DD. This configuration confers constitutive protease activity, enabling TRAF6/RIP1‐dependent activation of canonical NF‐κB signaling. CARD, caspase‐recruitment domain; DD, death domain; MAGUK, membrane‐associated guanylate kinase.

MALT1 is an 824 amino acid protein composed of five domains, including the N‐terminal death domain (DD), two immunoglobulin (Ig1/2) domains, the paracaspase domain, and the C‐terminal Ig3 domain (Figure 1A). The DD domain of MALT1 protein interacts with the caspase‐recruitment domain (CARD) of BCL‐10 protein to form the CARD‐BCL10‐MALT1 (CBM) complex, a critical assembly required for activation of the NF‐κB signaling pathway.^12^, ^13^ Concurrently, the Ig1 and Ig2 domains enhance the binding between MALT1 and BCL10 by interacting with the Ser/Thr‐rich domains adjacent to the CARD domain of BCL10.^14^ The paracaspase domain of MALT1, together with its Ig3 domain, is essential for MALT1's protease activity, enabling it to cleave specific sequence substrates and thereby participate a variety of physiological processes.^15^ Intracellularly, the MALT1 protein can be expressed in two alternative forms, MALT1A and MALT1B, with the MALT1B form lacking amino acids at positions 309–319, a region encoded by exon 7, which harbors the T6BM1 motif, that facilitates TRAF6 recruitment.^16^ TRAF6 binds to MALT1 through two key motifs: TDEAV**E**_**316**_CTE (aa 311–319) and PV**E**_**806**_TTD (aa 804–809; **E**_**806**_ in MALT1A, **E**_**795**_ in MALT1B).^16^, ^17^, ^18^ TRAF6, an E3 ubiquitin ligase, can ubiquitinate multiple substrate proteins such as IKKγ (NEMO), BCL‐10, MALT1, and itself to propagate downstream NF‐κB signaling.^19^

The *MALT1* gene was identified from the genome t (11; 18) (q21; q21) chromosomal translocation, which is highly prevalent in MALT lymphoma and results in the production of the oncogenic fusion protein API2‐MALT1. This fusion protein is the primary driver of sustained activation of the noncanonical NF‐κB signaling pathway in MALT lymphoma.^20^ The API2‐MALT1 fusion protein consists of three BIR domains of N‐terminal API2 and a MALT1 sequence excluding the DD domain, and its transcription is regulated by the API2 promoter ^21^(Figure 1B). Activation of API2‐MALT1 requires a dimerization process in which the first BIR domain of one fusion protein (BIR1) interacts with the paracaspase domain of another fusion protein. This dimerization allows API2‐MALT1 to recruit TRAF2 and receptor‐interacting protein‐1 (RIP1) in addition to TRAF6, which binds to the MALT1 molecule, thereby activating the classical NF‐κB pathway.^19^, ^22^

## 3 MALT1 AS A BIFUNCTIONAL REGULATOR OF NF‐κB SIGNALING: SCAFFOLD AND PROTEASE

### 3.1 Scaffold function of MALT1

The activation of MALT1 is intricately dependent on the CARD‐CC family proteins, including CARD9, CARD10 (CARMA3), CARD11 (CARMA1), and CARD14 (CARMA2), which are able to form distinct CBM complexes to regulate immune responses.^23^ CARD9, predominantly expressed in myeloid cells (e.g., macrophages and neutrophils), activates NF‐κB through signaling mechanisms of innate immune receptors such as TREM1 or Dectin‐1.^15^, ^24^ In contrast, CARD10 and CARD14 expression is mainly restricted to nonhematopoietic tissues. CARD14, enriched in keratinocytes, mediates NF‐κB activation through pattern recognition receptors (e.g., Dectin‐1),^25^ whereas CARD10, broadly expressed in the gut, heart, and kidney, is activated by the G protein‐coupled receptor (GPCRs) and epidermal growth factor receptors (EGFRs).^26^, ^27^, ^28^ CARD11 is expressed in B cells, T cells, NK cells, and mast cells, and is activated when the receptors on these cells (BCR, TCR, and NK receptors such as NKGD2, and mast cell FcεRI receptors) are stimulated by antigens.^29^, ^30^, ^31^ Among these pathways, TCR‐ and BCR‐mediated CARD11 activation mechanisms are best characterized, underscoring its pivotal role in immune signaling.

**FIGURE 2:**
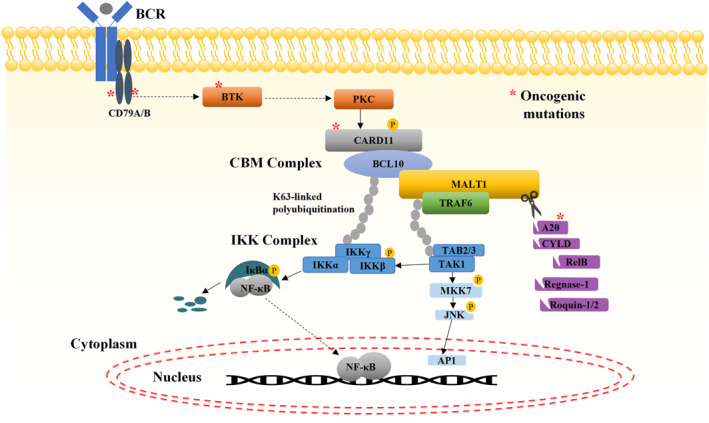
Dual Roles of MALT1 in B‐Cell Signaling. Antigen receptor triggering leads to the formation of the CBM complex, which acts as a recruiting platform for the ubiquitin ligase TRAF6, which mediates K63‐linked polyubiquitination of BCL10, MALT1, IKKγ (NEMO), and TRAF6 itself. The polyubiquitin chain of MALT1 and TRAF6 provides a scaffold for the recruitment of the kinase TAK1 through the TAB 2/3 adaptor protein, and the polyubiquitin chain of BCL10 promotes the recruitment of IKKγ/NEMO. IKK complex activation by TAK1 phosphorylates the IKKβ subunit, which phosphorylates the NF‐κB inhibitor IκBα to target it for proteasomal degradation, and subsequent NF‐κB nuclear translocation. Concurrently, TAK1 activates the AP‐1 pathway through MKK7‐JNK signaling. Within the CBM complex, MALT1 protease is activated, cleaving substrates involved in signaling and transcriptional and post‐transcriptional gene regulation such as A20, CYLD, RelB, Regnase‐1, and Roquin‐1/2. These cleavage events sustain NF‐κB activation and stabilize pro‐survival transcripts, amplifying B‐cell proliferation and immune responses.

In the resting state, CARD11 adopts an autoinhibited conformation stabilized by intramolecular interactions between the linker region, the CARD‐CC region, and the GUK‐SH3 domain.^32^ In T cells, antigen engagement triggers TCR signaling, leading to ZAP70‐dependent activation of protein kinase Cθ (PKCθ), which phosphorylates the CARD11 linker region.^33^ Subsequent phosphorylation by Akt and CAMK2 further relieves autoinhibition.^15^, ^34^ In B cells, SYK, and PKCβ phosphorylate and activate CARD11 upon BCR stimulation, enabling CC domain oligomerization and interaction with BCL10 through CARD–CARD domains.^28^, ^35^, ^36^ This interaction promotes BCL10 oligomerization to form fibril filaments, facilitating MALT1 recruitment through its N‐terminal DD. The C‐terminus of MALT1 protrudes beyond this helical core structure.^12^, ^37^ MALT1 acts as a scaffold in the CBM complex, recruiting E3 ubiquitin ligases such as TRAF6 and cIAP1/2, and the linear ubiquitin chain assembly complex LUBAC (composed of HOIL1, HOIP, and SHARPIN), mediating K63‐linked polyubiquitination of BCL10, MALT1, IKKγ (NEMO), and TRAF6.^13^, ^38^, ^39^ The polyubiquitin chain of MALT1 and TRAF6 provides a scaffold for the recruitment of kinase TAK1 through the TAB 2/3 adaptors.^23^ Concurrently, the IKK complex binds to the polyubiquitin chain of BCL10 through the IKKγ (NEMO) subunit, enabling TAK1‐dependent phosphorylation of IKKβ. Activated IKKβ phosphorylates IκBα, targeting it for βTrCP‐mediated proteasomal degradation, thereby releasing NF‐κB for nuclear translocation and transcriptional activation.^15^, ^39^ Additionally, CBM complex‐recruited TAK1 activates AP‐1 through the MKK7‐JNK pathway^54^, ^55^, ^56^ (Figure 2).

### 3.2 Protease function of MALT1

The paracaspase domain of MALT1 is highly homologous to mammalian capases and metacaspas in plants, fungi, and protozoa.^57^ However, it was not until 2008 that MALT1 was discovered as a cysteine protease.^58^, ^59^ Protease activation requires monoubiquitination at lysine 644 (K644) within the Ig3 domain, this post‐translational modification facilitates the dimerization of the paracaspase domain, a conformational change essential for forming the active protease state.^60^, ^61^ This dimerization enables substrate recognition—specifically, the substrate protein binds to a paracaspase domain‐specific groove, where the catalytic dyad (histidine 415 [His415] and cysteine 464 [Cys464]) cleaves peptide bonds exclusively at arginine (Arg) residues (rather than the aspartic acid [Asp] sites targeted by classical caspases).^15^, ^19^, ^59^ Structural studies reveal that His415 facilitates proton transfer to polarize the scissile bond, whereas Cys464 acts as the nucleophilic residue that attacks the carbonyl carbon, executing substrate cleavage.^19^ Computational and proteomic analyses predict approximately 30 potential MALT1 substrates, of which nearly 20 have been experimentally validated.^15^, ^62^ Common substrates for MALT1 are summarized in Table 1; typical substrates include signaling regulators, such as A20, CYLD, HOIL‐1, BCL10, and MALT1 itself. There are also proteins involved in transcriptional/post‐transcriptional modulators, such as RelB, Regnase‐1, and Roquin‐1/2. Cleavage of these substrates regulates immune signaling by sustaining NF‐κB activation and stabilizing pro‐inflammatory transcripts, thereby amplifying T‐l and B‐cell responses. These mechanisms not only elucidate MALT1's role in oncogenic signaling but also provide a structural basis for designing inhibitors targeting its protease activity. Although early strategies primarily explored competitive inhibition through interaction with the catalytic dyad, the identification of an allosteric pocket centered on residue E397 has enabled the development of potent and selective allosteric inhibitors. This was exemplified by the first co‐crystal structure of MALT1 in complex with thioridazine, which defined this conserved allosteric site as a preferred interface for effective and specific inhibition.^70^

**TABLE 1:** Summary of MALT1 protease substrates.

Substrate	Cleavage site	Outcome	Biological function	References
MALT1				
A20	GAS**R**_**439**_GEA	Inactivation	NF‐κB signaling pathway activation	^59^
CYLD	FMS**R**_**324**_GVG	Inactivation	JNK/NF‐κB signaling pathway activation	^63^
RelB	LVS**R**_**85**_GAA	Inactivation	NF‐κB signaling pathway activation	^5^
BCL10	LRS**R**_**228**_TVS	Inactivation, degradation	β1‐integrin‐mediated decline in T cell adhesion	^58^
HOIL‐1	LQP**R**_**165**_GPL	Inactivation	Downregulated of NF‐κB signaling pathway	^38^
MALT1	LCC**R**_**149**_ATG/	Dissociate from BCL10	BCL10‐independent NF‐κB signaling pathway activation	^17^, ^64^
MALT1A/MALT1B	HCS**R**_**781**_TPD/HCS**R**_**770**_TPD	Inactivation	Reduced proteolytic capacity of MALT1B; A limited effect on MALT1A	^17^
Regnase‐1	LVP**R**_**111**_GGG	Inactivation	mRNA stable (*c‐Rel*, *Ox40*, *IL‐2*).	^65^, ^66^
Roquin‐1	LIP**R**_**510**_GTD/MVP**R**_**579**_GSQ	Inactivation	mRNA stable (*IκBζ*, *IκBNS*, *IRF4*).	^66^
Roquin‐2	LIS**R**_**509**_TDS	Inactivation	mRNA stable (*IκBζ*, *IκBNS*, *IRF4*).	^66^
Tensin‐3	LVS**R**_**614**_CPA/AVQ**R**_**645**_GVG	Inactivation	B‐cell adhesion and decreased lymphoma spread	^67^
API2‐MALT1				
NIK	CLS**R**_**325**_GAH	Stabilization	Non‐canonical NF‐κB signaling pathway activation	^68^
LIMA1	PDS**R**_**206**_ASS/FKS**K**_**289**_GNY	Inactivated, oncoproteins are generated	MALT lymphoma oncogenesis	^69^

The API2‐MALT1 fusion protein retains the canonical substrate specificity of native MALT1 for A20 and CYLD but exhibits unique proteolytic activity toward atypical substrates, including NIK and LIMA1, through its BIR domain‐mediated recruitment mechanism^59^, ^62^, ^71^ (Table 1). This aberrant cleavage spectrum drives constitutive NF‐κB activation and oncogenic transformation in MALT lymphoma.

Although MALT1 scaffolding is indispensable for initial NF‐κB activation upon antigen stimulation, its protease activity is critical for sustained signaling through the cleavage of negative regulators (e.g., A20, CYLD, RelB). Genetic studies in MALT1‐knockout mice or those expressing the catalytically inactive mutant (MALT1 C472A) reveal severe defects in B1 cells, marginal zone B cells, and regulatory T cells (Tregs).^72^, ^73^, ^74^, ^75^ These mice exhibited an attenuated immune response to antigen stimulation, decreased immunoglobulin levels in vivo, and mixed inflammatory cell infiltration in multiple organs. These findings highlight the importance of the protease function of MALT1 in maintaining immune cell development and functional integrity.

## 4 ONCOGENIC ROLES OF MALT1 ACROSS TUMOR TYPES

The oncogenic potential of MALT1 is most definitively demonstrated in the context of B‐cell malignancies, where its activation is frequently driven by specific genetic lesions or chronic signaling pathways. In these cancers, MALT1 functions as a central signaling node, promoting cell survival, proliferation, and immune evasion through both scaffold‐dependent and protease‐dependent mechanisms. In solid tumors, the evidence is more nuanced and context‐dependent; although MALT1 is often overexpressed and contributes to oncogenic phenotypes in experimental models, its role is typically that of a key amplifier of upstream oncogenic signals rather than a primary driver. The following sections detail the mechanisms in specific cancer types, highlighting the robust evidence in lymphomas and the emerging, though sometimes correlative, data in solid tumors.

### 4.1 Hematologic tumors

#### 4.1.1 Role of MALT1 in diffuse large B‐cell lymphoma (DLBCL)

The BCR signaling is central to B‐lymphocyte maturation, survival, and malignant transformation, driving the pathogenesis of hematologic malignancies, such as non‐Hodgkin's lymphoma (NHL), DLBCL, chronic lymphocytic leukemia (CLL), mantle cell lymphoma (MCL).^37^ Key regulators of BCR pathway, such as spleen tyrosine kinase (SYK), Bruton tyrosine kinase (BTK), phosphatidylinositol (PI) 3‐kinase (PI3K), and PKCβ, are promising therapeutic targets. Inhibition strategies against these targets open new avenues for precision medicine.^32^, ^76^ The BTK inhibitor ibrutinib has shown significant clinical efficacy in MCL and CLL.^77^, ^78^ In addition, the combination of ibrutinib and chemoimmunotherapy R‐CHOP improves survival of patients with DLBCL harboring the BCR receptor CD79B mutation.^79^ However, in patients with CARD11 gain‐of‐function mutations, ibrutinib is less effective.^80^, ^81^ Furthermore, ibrutinib treatment may induce mutations at the BTK C481 locus, leading to drug resistance and limits its long‐term efficacy, underscoring the need for alternative strategies.

DLBCL, the most prevalent NHL subtype (35% of cases), exhibits marked genetic diversity. Molecular stratification identifies three subtypes, namely activated B‐cell, germinal center B‐cell, and primary mediastinal B‐cell lymphoma (PLBL).^82^ ABC‐DLBCL subtype is more aggressive, characterized by chronic NF‐κB activation, has a 3‐year progression‐free survival rate of only 40% with standard R‐CHOP chemoimmunotherapy.^83^ Oncogenic mutations in *CD7*9A/B, *CARD11*, *MYD88*, *TNFAIP3* (A20), *PLCγ1*, and *PKCβ* drive constitutive BCR and TLR signaling in ∼20% of ABC‐DLBCL cases.^39^, ^81^, ^84^, ^85^ Although BTK inhibitors (e.g., ibrutinib) and PKCβ inhibitors (e.g., sotrastaurin) show efficacy in CD79A/B‐mutant tumors,^79^, ^80^, ^85^, ^86^ *CARD11* mutations confer resistance by enabling CBM complex formation independent of upstream signals.^32^, ^80^ Co‐occurring *MYD88* mutations further amplify NF‐κB activation, highlighting the complexity of ABC‐DLBCL pathogenesis.^87^ The CBM complex is indispensable for ABC‐DLBCL survival, with genetic or pharmacological inactivation of CARD11, BCL10, and MALT1 potently suppressing ABC‐DLBCL cell proliferation.^4^, ^88^, ^89^ Therefore, CBM complex molecules are not only key to the survival of chronic BCR and ABC‐DLBCL, but also a potential target for anticancer therapy, especially for patients who are ineffective or have developed resistance to existing BCR signaling drugs.

MALT1 drives the oncogenesis of ABC‐DLBCL through a multifaceted mechanisms, which can be categorized into several distinct pathways: (1) transcriptional control: MALT1 enhances the canonical NF‐κB signaling pathway, leading to the transcriptional upregulation of genes that promote cell survival and proliferation. (2) Post‐transcriptional control: Through its protease activity, MALT1 cleaves RNA‐binding proteins such as Regnase‐1 and Roquin‐1/2. This prevents the degradation of key mRNAs (e.g., NFKBIZ, NFKBID), thereby stabilizing transcripts that sustain NF‐κB activation and inflammatory signaling.^71^ (3) Post‐translational modifications: MALT1 itself is regulated by phosphorylation; for instance, CK1α‐mediated phosphorylation at serine 562 enhances its function and promotes ABC‐DLBCL proliferation. Furthermore, the critical role of protease activity is underscored by mutations such as K644R, which disrupts ubiquitination‐dependent activation, and the catalytic dead mutant C464A, both of which impair ABC‐DLBCL survival.^29^, ^61^ (4) Metabolic reprogramming: MALT1 protease activity fosters a metabolic shift by enhancing glutamine uptake and promoting the expression of immune checkpoint molecules such as PD‐L1, which facilitates tumor immune evasion.^28^, ^90^ (5) Malignant progression: MALT1 inhibition has been shown to prevent the transformation of p53‐deficient MALT lymphomas into more aggressive ABC‐DLBCL, highlighting its role in malignant lineage transition.^91^ The multifunctionality of MALT1 establishes it as a compelling therapeutic target. Its inhibition concurrently addresses the chronic activation of the NF‐κB pathway and counteracts tumor immune evasion, as well as overcome BTK‐targeted therapies.

#### 4.1.2 MALT lymphoma

Patients with MALT1 lymphoma account for about 8% of NHL, with a predilection for gastric mucosa. *Helicobacter pylori* infection is strongly implicated in its pathogenesis, driving chronic inflammation through pro‐inflammatory cytokine release and neutrophil infiltration. This inflammatory milieu induces genomic instability, facilitating the t (11; 18) (q21; q21) chromosomal translocation, resulting in the production of oncogenic API2‐MALT1 fusion protein, which exhibits constitutive protease activity.^20^, ^22^, ^92^ API2‐MALT1 cleaves NF‐κB‐inducing kinase (NIK), stabilizing it to activate the noncanonical NF‐κB pathway through IKKα‐mediated phosphorylation of p100, yielding transcriptionally active p52.^68^ Concurrently, API2‐MALT1 proteolyzes LIMA1, a tumor suppressor, disrupting its LIM domain‐dependent antiproliferative and antiadhesive functions.^69^ Additionally, a mutually exclusive t (14; 18) (q32; q21) translocation, present in 15%–20% of cases, drives MALT1 overexpression under the IgH promoter, further amplifying NF‐κB signaling.^93^

#### 4.1.3 MALT1 and other hematologic tumors

The protease function of MALT1 is indispensable for the growth and proliferation of a variety of B‐cell malignancies. Pharmacological inhibition of MALT1 significantly suppresses proliferation of MCL and CLL cells in preclinical models.^94^, ^95^ In multiple myeloma (MM), elevated MALT1 expression correlates with poor prognosis. Genetic ablation of MALT1 inhibits MM cell proliferation, clonogenicity, and in vivo tumor growth.^96^ In addition, MALT1 deficiency or protease inhibition abrogates NF‐κB activation and proliferation in adult T‐cell leukemia cell lines, highlighting its role in T‐cell malignancies.^97^ These findings highlight MALT1 as a pan‐hematologic malignancy target, with therapeutic potential extending beyond B‐cell disorders to T‐cell neoplasms.

### 4.2 Solid tumors

The critical role of CBM complexes in hematologic malignancies has been well‐established. Recent studies have further elucidated the activation of MALT1 in various solid tumors, involving melanoma,^98^ hepatocarcinoma,^99^, ^100^ lung cancer,^101^, ^102^ breast cancer,^103^, ^104^ glioblastoma,^105^, ^106^ pancreatic carcinoma,^107^ and prostate cancer.^108^ In metastatic melanoma, elevated MALT1 expression enhances cell viability through the activation NF‐κB and JNK signaling pathways.^98^ In hepatocellular carcinoma cells, high MALT1 levels suppress apoptosis by competing with TIFA for binding to TRAF6.^100^ In lung cancer, MALT1 facilitates the activation of NF‐κB and STAT3 signaling driven by EGFR mutations, which are critical for tumor progression.^101^ In breast cancer, the overexpression of GPCR receptors AT1R and PAR1 is associated with metastasis and poor prognosis. The CBM complex exacerbates this process by mediating PAR1‐induced NF‐κB activation, thereby promoting cell proliferation and metastasis.^103^ In glioblastoma, MALT1 regulates the autophagy and death of cancer stem cell‐like cells by modulating lysosomal abundance. Notably, the absence or inhibition of MALT leads to an increase in lysosomal abundance, which in turn induces autophagy and death.^105^, ^106^ Similarly, studies in pancreatic carcinoma and prostate cancer have demonstrated that high MALT1 expression is a central driver of NF‐κB‐mediated cancer progression. Silencing or pharmacological inhibition of MALT1 effectively inhibits cell proliferation and aggressiveness in these cancers.^107^, ^108^ These findings collectively highlight the CBM complex as a key signaling node in the pathogenic activation of NF‐κB. By regulating cell proliferation, survival, invasion, migration, and metastasis, MALT1 plays a pivotal role in tumor initiation and progression. Therefore, targeting MALT1 represents a promising therapeutic strategy for the treatment of solid tumors.

### 4.3 Function of MALT1 in the tumor immune microenvironment

MALT1 is an immunomodulatory protease with dual roles in the tumor biology, directly promoting tumor cell survival and proliferation while simultaneously shaping the immunosuppressive tumor microenvironment (TME) through its enzymatic activity. A key mechanism involves its regulation of regulatory T cells (Tregs), which maintain immune tolerance by suppressing antitumor immunity. MALT1 protease activity is critical for sustaining the immunosuppressive phenotype of Tregs, particularly their Foxp3 expression and functional integrity.^75^, ^109^, ^110^ Pharmacological inhibition of MALT1 reprograms tumor‐infiltrating Tregs, converting them from immunosuppressive Foxp3^+^ cells into pro‐inflammatory effector cells. This phenotypic shift disrupts the immunosuppressive TME, induces localized inflammatory responses, and synergizes with immune checkpoint inhibitors (e.g., anti‐PD‐1 antibodies) to enhance antitumor immunity.^8^ Concomitantly, MALT1 inhibition amplifies the effector functions of CD4^+^ and CD8^+^ T cells by attenuating Treg‐mediated suppression, thereby increasing interferon‐γ (IFNγ) production and cytotoxic activity.^75^ However, this therapeutic strategy may inadvertently upregulate PD‐L1 expression on tumor cells through enhanced Th1‐driven inflammation, potentially limiting efficacy through PD‐1/PD‐L1 axis reactivation.^109^ These findings underscore the dual nature of MALT1 in tumor progression: it sustains both intrinsic oncogenic signaling and extrinsic immune evasion. Therefore, combining MALT1 inhibitors with PD‐1/PD‐L1 blockade presents a rational strategy to counteract compensatory immune resistance mechanisms. Preclinical studies suggest that such combinations may overcome PD‐L1‐mediated adaptive resistance while leveraging MALT1 inhibition to remodel the TME, offering a promising approach for tumors reliant on immunosuppressive networks.

## 5 PROGRESS IN THE DEVELOPMENT OF MALT1 INHIBITORS

The discovery of MALT1 protease activity has paved way for development of potent and selective inhibitors as potential anticancer agents or immunomodulators.^11^ Early studies utilized irreversible peptides, such as zVRPR‐fmk, or peptidomimetic‐like inhibitors, which covalently bind to the catalytic centers of MALT1 to elucidate its biological functions in immune and cancer cells.^4^, ^7^, ^89^ However, these peptide‐derived compounds face significant limitations in clinical development due to insufficient selectivity, poor cellular uptake, and short in vivo half‐lives. The small molecule nonpeptide compound MI‐2 induces ABC‐DLBCL cell death in vitro and in vivo by forming irreversible covalent bonds with MALT1 catalyzed cysteine, but its clinical application is hindered by its limited potency and specificity.^111^, ^112^ Furthermore, recent evidence indicates that MI‐2 directly inhibits GPX4, leading to ferroptosis induction independently of MALT1 or RC3H1.^113^ Thus, MI‐2 acts primarily through GPX4 inhibition rather than specific MALT1 blockade, necessitating caution in interpreting data generated with this compound.

Current MALT1 inhibitors in preclinical or clinical development primarily function by interfering with MALT1 protease activity. Among these, benzothiazide derivatives, such as mepazine and thioridazine, represent the first class of compounds that inhibit MALT1 in a non‐competitive manner by binding to the allosteric pocket located between the paracaspase and Ig3 domains.^6^ These compounds bind specifically to a structurally conserved allosteric site centered on residue E397, which is critical for maintaining MALT1's catalytically active conformation. Structural studies revealed that binding of mepazine or thioridazine to the E397 site induces a conformational lock in the Ig3 domain, preventing the dynamic rearrangement required for protease activation.^70^ Compared to competitive inhibitors targeting the catalytic site, allosteric inhibitors exhibit superior selectivity and improved pharmacological properties. Although mepazine has been shown to interact with other nonprotease targets,^114^ its favorable drug‐like properties have enabled its successfully application in a variety of preclinical cancer models driven by intracellular or extracellular MALT1 activity.^6^, ^75^, ^106^, ^109^ These findings demonstrate the therapeutic potential of allosteric MALT1 inhibition as a precision strategy to selectively suppress pathogenic MALT1 activity. However, it is important to note that allosteric inhibitors also affect physiological protease functions, including Treg development and function, as evidenced by Treg reduction in Malt1 paracaspase‐deficient mice and pharmacological studies.^72^, ^73^, ^74^, ^115^

Based on the structural insights into MALT1, series of allosteric MALT1 inhibitors with higher potency and selectivity have been developed.^11^, ^116^, ^117^ For instance, the MLT compounds developed by Novartis, including the lead compounds MLT‐943 and MLT‐985, have demonstrated potent inhibition of MALT1 protease activity. These compounds exhibit improved metabolic stability and achieve near‐complete inhibition of MALT1 protease activity in preclinical models of B‐cell lymphoma.^50^, ^115^, ^118^ However, it should be noted that Novartis has also reported severe Treg cell depletion and IPEX‐like syndrome under long‐term MALT1 inhibition in animal models, highlighting a significant challenge for the clinical translation of such compounds.^115^ As of now, clinical trials for these agents have not been announced.

Johnson & Johnson's MALT1 inhibitor, safimaltib (JNJ‐67856633), has demonstrated promising activity in preclinical tumor models of ABC‐DLBCL.^119^, ^120^, ^121^ Johnson & Johnson has initiated three clinical trials evaluating safimaltib for the treatment of relapsed/refractory NHL and CLL, both as monotherapy or in combination with the BTK inhibitor ibrutinib (NCT03900598, NCT04657224 and NCT04876092). In 2023, Johnson & Johnson announced the results of a Phase I clinical trial (NCT04876092) of safimaltib in NHL patients who had received at least one or two prior lines of therapy. Among 36 patients treated with the recommended phase II dose (RP2D), the objective response rate (ORR) was 27.8%, with 11.1% of patients achieving a complete response. The most common treatment‐emergent adverse events (TEAT) were mainly hyperbilirubinemia (44%), anemia (35.8%), and neutropenia (32.1%).^9^ These results provide clinical proof of concept for MALT1 inhibition as a targeted anticancer strategy. However, the observed toxicity profile—particularly the high incidence of hematologic adverse events—underscores the need for careful safety management and monitoring of long‐term hematologic and immune‐related toxicities in future clinical studies.

SGR‐1505, discovered using Schrödinger's proprietary computational platform, entered Phase I clinical trial (NCT05544019) after demonstrating potent inhibition of MALT1 activity and antiproliferative effects in both BTKi‐sensitive and resistant ABC‐DLBCL cell lines, as well as tumor‐suppressive activity in xenograft models.^41^ In the first‐in‐human Phase I trial of SGR‐1505, 33 patients with relapsed/refractory B‐cell malignancies received doses ranging from 50 to 300 mg once daily or 50–150 mg twice daily. Treatment was well‐tolerated, with no dose‐limiting toxicities and common G3/4 TEAEs included neutrophil count decreased (21%) with no febrile neutropenia. ORR were observed in 5 of 23 evaluable patients, as well as partial and minor responses in CLL/SLL and Waldenström macroglobulinemia, including patients previously exposed to both BTK and BCL2 inhibitors. These results support further development of SGR‐1505 as a well‐tolerated and biologically active MALT1 inhibitor with promising monotherapy efficacy.^122^

In addition to Johnson & Johnson and Schrödinger, other pharmaceutical companies are actively pursuing MALT1‐targeted therapies (Table 2). Several pharmaceutical companies progressed MALT1 inhibitors into clinical trials. AbbVie initiated Phase I trials for ABBV‐525 (NCT05618028), which showed strong MALT1 inhibition, suppression of B‐cell receptor signaling, and antitumor efficacy in BTKi‐resistant models, both as monotherapy and in combination with venetoclax.^45^ Ono Pharmaceuticals advanced ONO‐7018 into Phase I trials (NCT05515406), highlighting its ability to inhibit BTKi‐resistant cell proliferation and synergize with tirabrutinib in DLBCL and MCL models.^43^ Exscientia initiated Phase I trials for EXS73565 (NCT06980116), a selective MALT1 inhibitor designed using generative AI and molecular dynamics. The agent potently suppresses ABC‐DLBCL cell proliferation and drives durable tumor regression in patient‐derived xenografts; its activity is further enhanced when combined with ibrutinib, suggesting a strategy to overcome resistance.^48^ Aurigene also advanced AUR‐112, an orally available allosteric inhibitor engineered for low plasma protein binding and nanomolar‐level potency with strong whole‐blood activity in NF‐κB‐driven ABC‐DLBCL models, into a first‐in‐human Phase I trial (NCT067554506).^49^ Currently, no clinical data for these inhibitors are currently available. Furthermore, Monopteros Therapeutics is investigating the clinical safety and efficacy of the MALT1 inhibitor MPT‐0118, for tumor‐cell extrinsic proinflammatory reprogramming of Treg cells in the TME to boost antitumor immunity as a single agent monotherapy or in combination with the anti‐PD‐1 antibody pembrolizumab in advanced and refractory solid tumors (NCT04859777).^123^ These programs highlight diverse strategies to harness MALT1 inhibition for oncology applications, particularly in overcoming resistance and enhancing antitumor immunity. Ongoing trials will clarify their safety profiles, efficacy in resistant populations, and optimal combination strategies, paving the way for MALT1‐targeted therapies.

**TABLE 2:** Summary of MALT1 inhibitors.

Compound	Structure	Company	In vitro potency, IC_50_ (nM)		Phase	Indications	References
			MALT1 protease	Cellular viability			
Safimaltib (JNJ‐67856633)	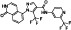	Janssen	74 ± 23	OCI‐Ly3: 496 ± 262; OCI‐Ly10: 332; TMD8: 635; HBL1: 547	Phase I (NCT03900598)	R/R NHL and CLL	^9^, ^40^
SGR‐1505	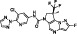	Schrödinger	1.3	OCI‐Ly10: 71	Phase I (NCT05544019)	B‐cell malignancy	^41^, ^42^
ONO‐7018	Undisclosed	ONO Pharmaceuticals	/	/	Phase I (NCT05515406)	R/R NHL and CLL	^43^, ^44^
ABBV‐525	Undisclosed	Abbvie	/	/	Phase I (NCT05618028)	B‐cell malignancy	^45^
MTP‐0118	Undisclosed	Monopteros therapeutics	/	/	Phase I (NCT04859777)	Solid tumors	^46^
XL114	Undisclosed	Aurigene oncology	/		Phase I (NCT05144347)	NHL	^47^
EXS73565	Undisclosed	Exscientia	<100	OCI‐Ly3:<400; TMD8: <300;	Phase I (NCT06980116)	R/R B‐cell malignancy	^48^
AUR112	Undisclosed	Aurigene	/	/	Phase I (NCT06755450)	Relapsed advanced lymphoma	^49^
MLT‐943	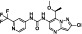	Novartis	4	/	Preclinical	/	^50^
BPI‐530616	Undisclosed	Betta	2	OCI‐Ly3: 98	Preclinical	/	^51^
HST‐1021	Undisclosed	Hotspot therapeutics	/	/	Preclinical	/	^52^
SY‐12696	Undisclosed	Shouyao holdings	3.73	OCI‐Ly3: 220	Preclinical	/	^53^

Abbreviations: R/R NHL and CLL, Relapsed/Refractory non‐Hodgkin's lymphoma and chronic lymphocytic leukemia.

## 6 SUMMARY AND OUTLOOK

The potential of MALT1 as a therapeutic target in cancer is gaining increasing recognition, and the development of MALT1 inhibitors offers new promise for cancer therapy. The mechanism of action of MALT1 across various tumor types suggests that MALT1 inhibitors may hold therapeutic potential in both hematologic and solid tumors, particularly in cases where tumors exhibit resistance to existing treatments. MALT1 inhibitors have the potential to simultaneously modulate tumor cell growth and immune cell function within the tumor microenvironment, providing a novel strategy that integrates precision oncology with immunotherapy. Clinical trials of MALT1 inhibitors are currently underway to evaluate the safety and efficacy of these inhibitors in the treatment of malignant lymphomas and solid tumors.

However, the development process of MALT1 inhibitors also faces many challenges, and several critical considerations must be addressed: (1) specificity and selectivity: Achieving high selectivity for MALT1 is essential to minimize off‐target effects on structurally related proteases and immune signaling pathways. Rational drug design, particularly through exploitation of the unique allosteric pocket surrounding E397, offers a promising route to enhance specificity. Allosteric inhibitors stabilize the inactive conformation of MALT1, thereby conferring selectivity over canonical cysteine proteases and reducing the risk of unintended pathway modulation. Structural optimization and allosteric targeting strategies may enhance selectivity. (2) Dosing strategy to limit autoimmune toxicity: Given the central role of MALT1 in regulatory Treg function, dosing regimens must be optimized to avoid excessive immunosuppression. Preclinical studies indicate that continuous MALT1 inhibition can deplete Tregs and provoke IPEX‐like syndromes. Intermittent dosing or adaptive titration protocols may help preserve Treg‐mediated immune homeostasis while maintaining antitumor efficacy. (3) Effects of long‐term inhibition: Prolonged MALT1 inhibition may lead to Treg cell depletion and autoimmune toxicity, necessitating close monitoring in clinical applications to ensure patient safety and establish safe therapeutic windows. (4) Biomarker‐driven patient stratification: Identifying predictive biomarkers is crucial for patient stratification and personalized therapy. Biomarkers may include genetic alterations (e.g., *CARD11* mutations), NF‐κB pathway activation signatures, including elevated expression of NF‐κB target genes (e.g., NFKBIZ and NFKBID) or nuclear localization of p65, or immune cell profiling in the TME. Through integrated approaches in drug design, clinical dosing, safety monitoring, and patient stratification, the translational potential of MALT1 inhibitors can be fully realized while mitigating their unique immunological risks.

The development of MALT1 inhibitors represents a field replete with both challenges and opportunities. As clinical trials advance, further insights into the safety, efficacy, and optimal clinical applications of these inhibitors will emerge. Strategic combination therapies, such as pairing MALT1 inhibitors with immune checkpoint blockade or targeted agents, may enhance antitumor activity while overcoming resistance mechanisms. With meticulous design and rigorous evaluation, MALT1 inhibitors have the potential to become a cornerstone in the arsenal of cancer therapies, offering novel treatment options for patients in need.

## AUTHOR CONTRIBUTIONS

The authors confirm contribution to the paper as follows: Xintao Cao and Peixia Wang: Conceptualization, Methodology, Writing—Original Draft. Yuan Ji and Yongliang Sun: Investigation, Resources, Validation. Dejiu Zhang: Supervision, Writing—Review & Editing. All authors reviewed the results and approved the final version of the manuscript.

## CONFLICT OF INTEREST STATEMENT

The authors declare no conflicts of interest.

## ETHICS STATEMENT

Not applicable.

## Data Availability

The data generated and analyzed during the current study are available from the corresponding author upon reasonable request.
